# Dartos flap rotation for severe congenital penile torsion in a nine-year-old: a case report and surgical insights

**DOI:** 10.1093/jscr/rjaf164

**Published:** 2025-03-27

**Authors:** Isaack Mlatie, Ibrahim Ndola, Alfred Chibwae, Shukrani Ngereja, Rajabu Bakari, Charles Nhungo, Fransia Arda, Obadia Nyongole

**Affiliations:** Department of Surgery, School of Medicine, Muhimbili University of Health and Allied Sciences, Dar es Salaam, Tanzania; Department of Urology, Muhimbili National Hospital, Dar es salaam, Tanzania; Department of Urology, Muhimbili National Hospital, Dar es salaam, Tanzania; Department of Urology, Muhimbili National Hospital, Dar es salaam, Tanzania; Department of Urology, Muhimbili National Hospital, Dar es salaam, Tanzania; Department of Urology, Muhimbili National Hospital, Dar es salaam, Tanzania; Department of Urology, Muhimbili National Hospital, Dar es salaam, Tanzania; Department of Urology, Muhimbili National Hospital, Dar es salaam, Tanzania

**Keywords:** penile torsion, Dartos fascia flap, isolated severe penile torsion

## Abstract

Congenital penile torsion, a rare anomaly characterized by the rotation of the penile shaft along its longitudinal axis, often goes unrecognized in low- and middle-income countries. This case highlights the management of a nine-year-old male presenting with isolated severe penile torsion using a Dartos flap rotation technique. The surgical approach was successful, with no postoperative complications, underscoring its utility as a simple and effective method, particularly in resource-limited settings.

## Introduction

Congenital penile torsion is an uncommon congenital condition involving the axial rotation of the penis, typically in a counterclockwise direction. Its severity ranges from mild (30°) to severe (>90°) and may occur in isolation or alongside other anomalies such as hypospadias. Despite its cosmetic and potential functional implications, penile torsion remains underreported, especially in LMICs. Surgical correction is typically indicated for torsions exceeding 60°, primarily for cosmetic reasons and to prevent long-term functional sequelae.

Worldwide, the true incidence of congenital penile torsion is unknown, but isolated cases are reported in 1.7%–27% of neonates, with torsions greater than 90° seen in 0.7% of cases [1]. Long-term functional problems, particularly in adulthood, have yet to be clearly established. This case report presents the successful surgical management of isolated severe congenital penile torsion in a nine-year-old, employing a Dartos flap rotation technique. The study aims to contribute to the limited body of literature on this condition and highlight an effective surgical approach suitable for LMIC settings.

## Case presentation

A nine-year-old male presented with a congenital left-sided penile torsion associated with a left-directed urine stream, causing frequent wetting of the left thigh during urination. He reported no history of urinary tract infections, incontinence, or weak urine stream. The condition persisted despite circumcision performed at age two.

**Figure 1:**
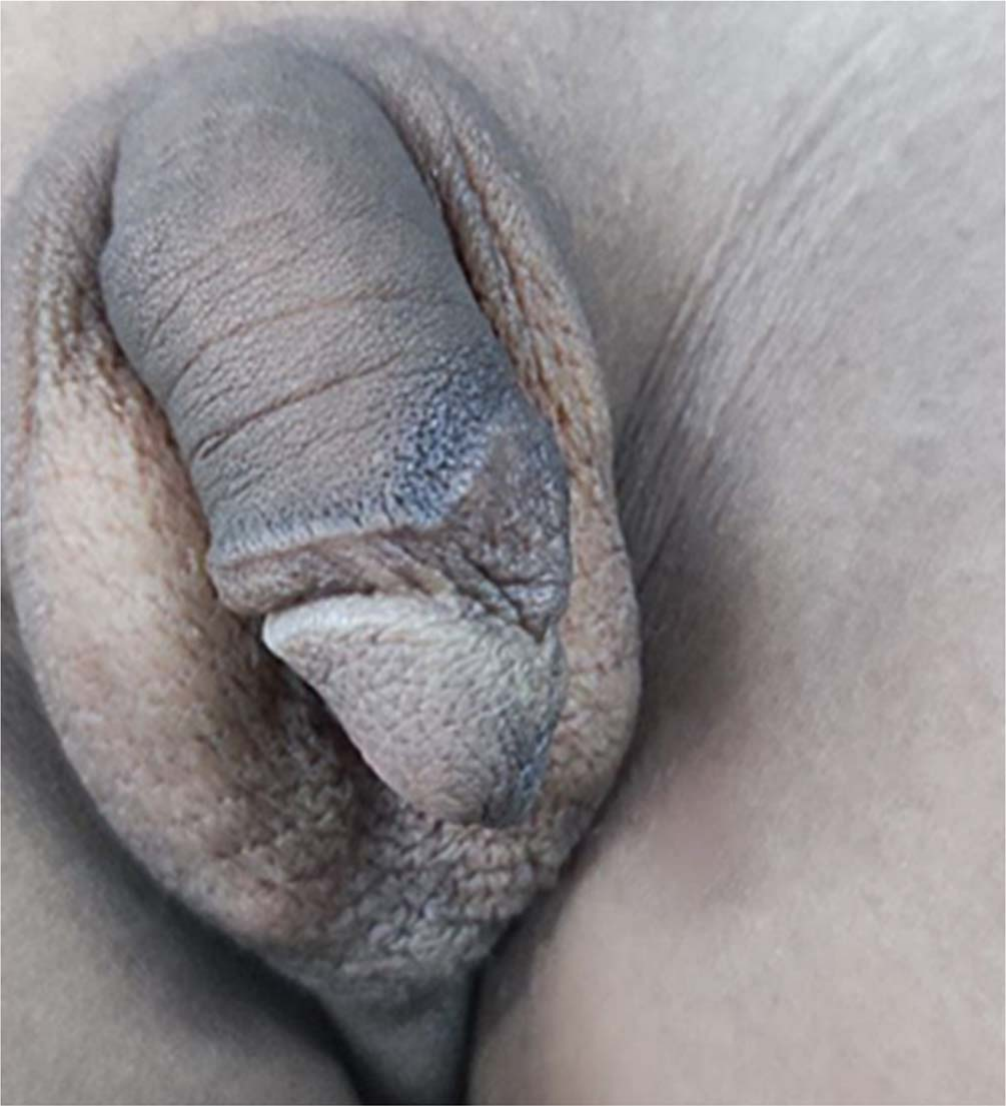
An image showing a counter-clockwise rotated penis to the left side >90° from the scrotal median raphe.

Physical examination revealed a circumcised penis of normal size with a counterclockwise torsion exceeding 90° (Fig. 1). The scrotal skin and testicular examination were unremarkable. A diagnosis of congenital penile torsion was established, and surgical correction was planned.

### Surgical technique

**Figure 2:**
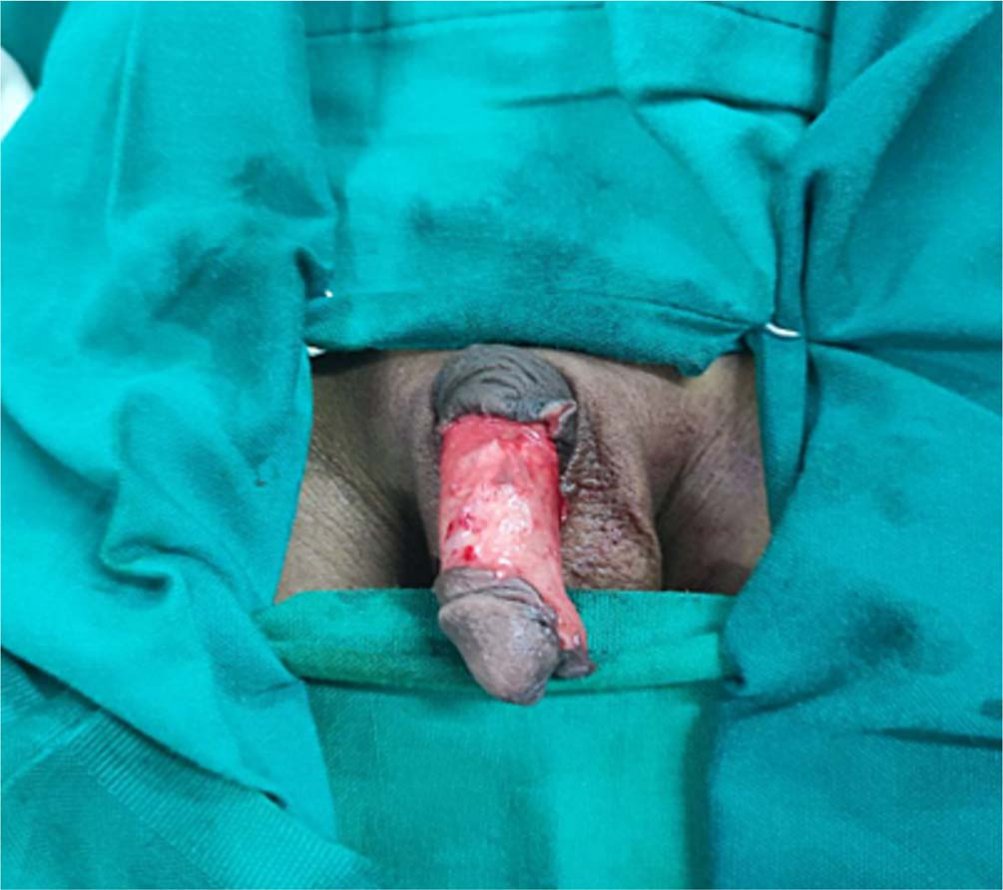
An image showing a circumferential incision a few millimeters proximal to the coronal sulcus with degloved penile skin up to the penile base.

**Figure 3:**
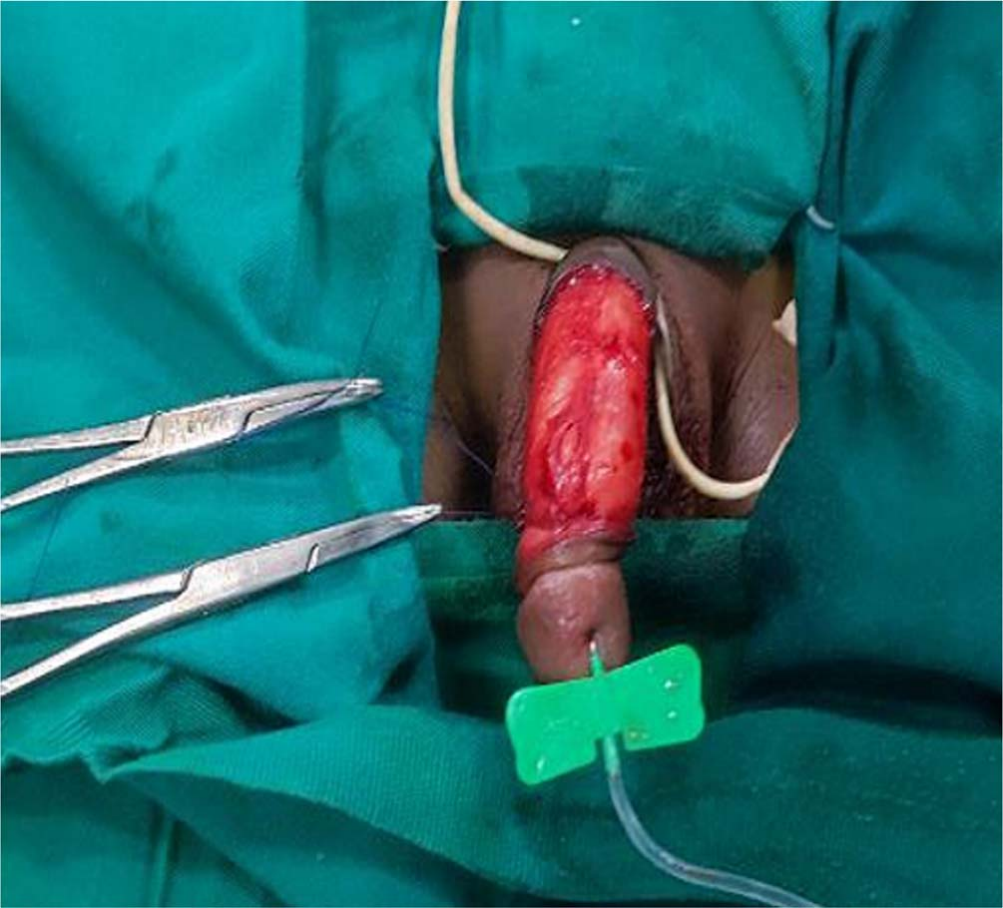
Artificial penile erection using a butterfly needle to assess the correction of the torsion by penile skin degloving.

**Figure 4:**
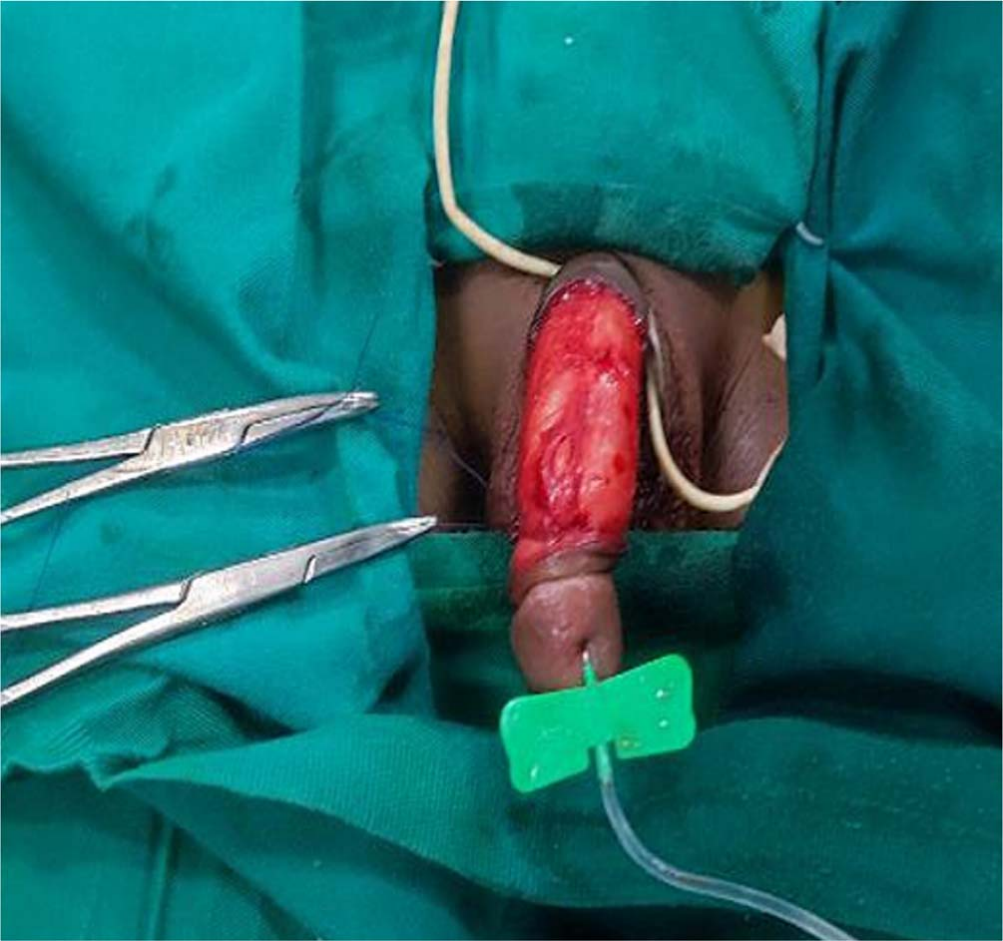
Rotated flap around the penis against the direction of penile torsion then sutured to the ventral aspect of the shaft of the corpus cavernosum.

Under general anesthesia, a circumferential incision was made proximal to the coronal sulcus, followed by degloving of the penile skin to the penile base (Fig. 2). An artificial erection (Gittes test) confirmed the persistence of torsion after degloving (Fig. 3). A dorsal Dartos flap was created and rotated against the direction of torsion. The flap was sutured to the ventral shaft of the corpus cavernosum using absorbable sutures (PDS 5/0) (Fig. 4).

**Figure 5:**
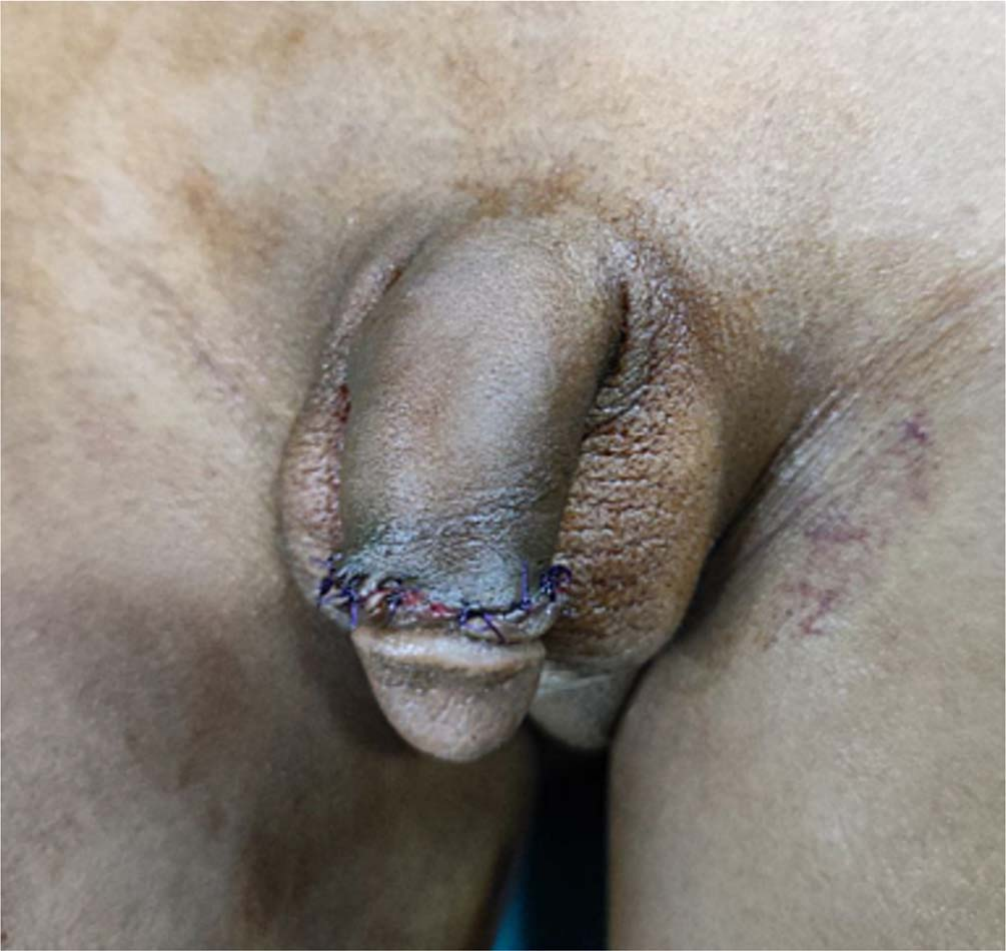
An image showing a skin overcorrection and skin realignment with some degree of over-rotation against the direction of residual penile rotation.

**Figure 6:**
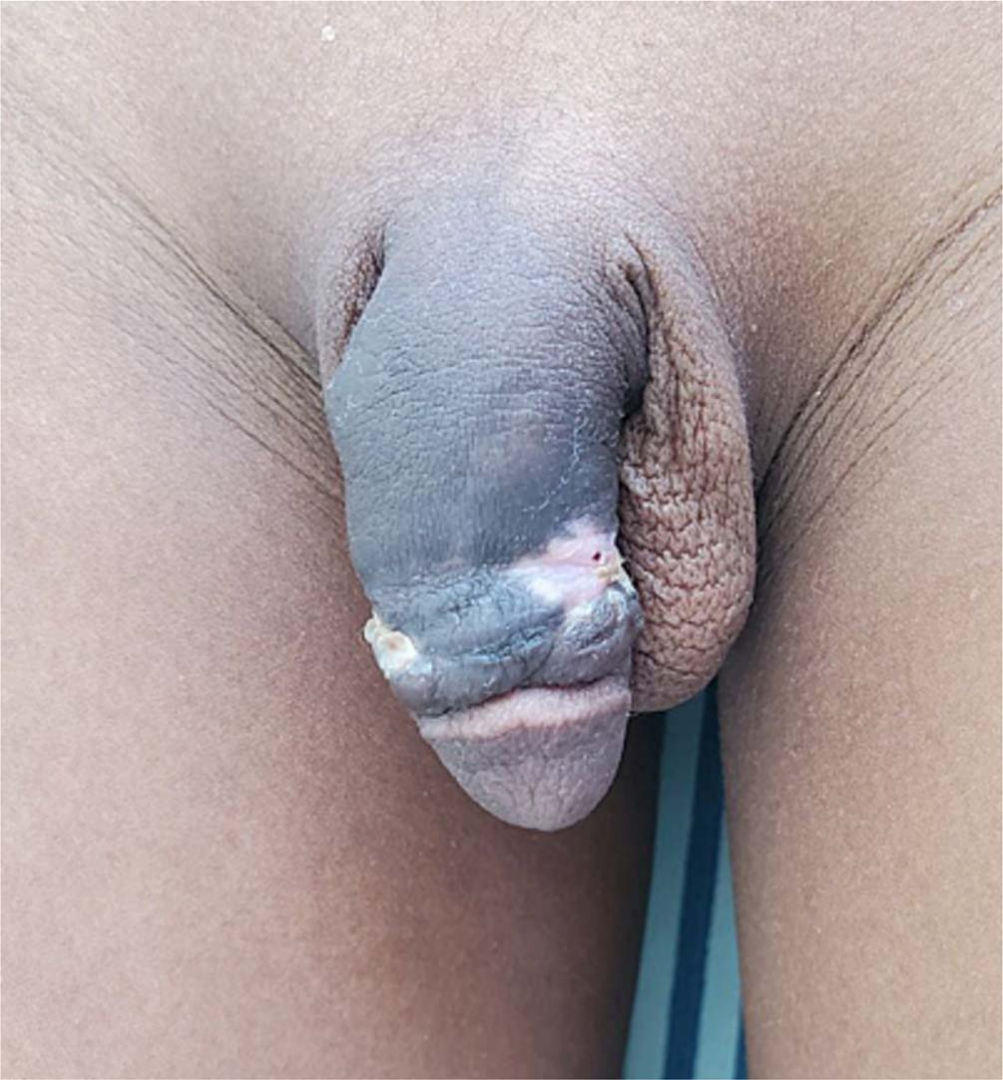
An image at fourth week postoperation showing a healed and corrected congenital penile torsion.

**Figure 7:**
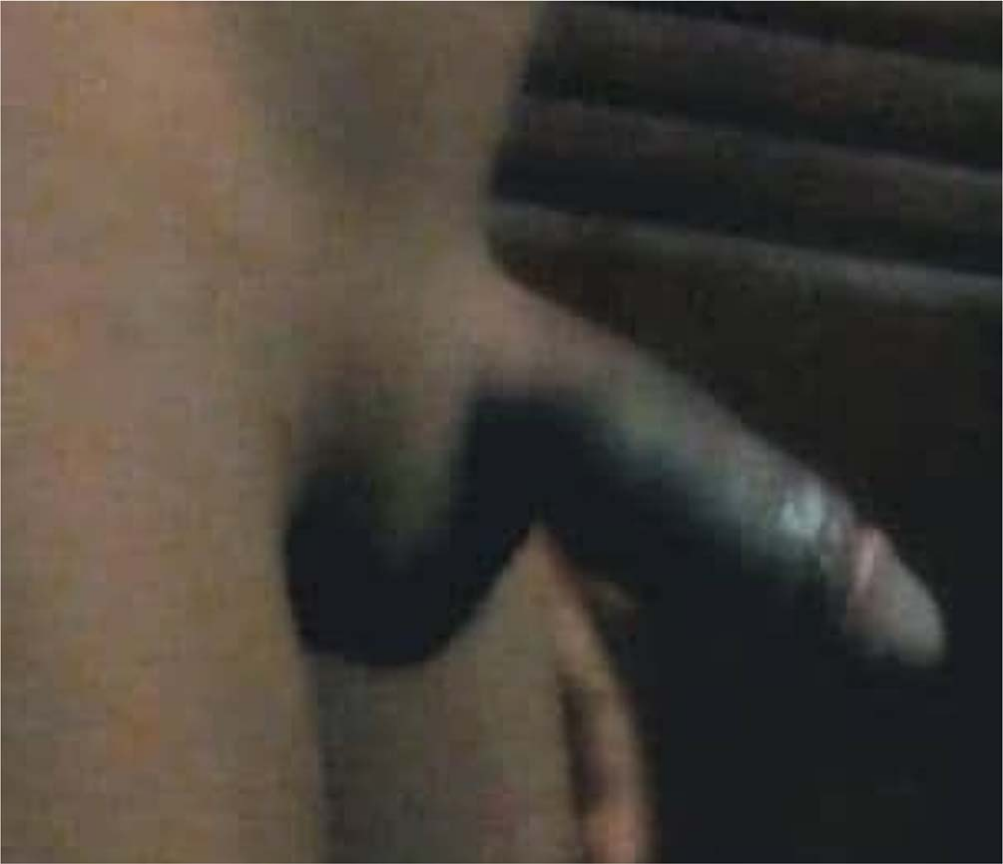
An image at 3 months follow-up visit showing an erect image of corrected congenital penile torsion.

**Figure 8:**
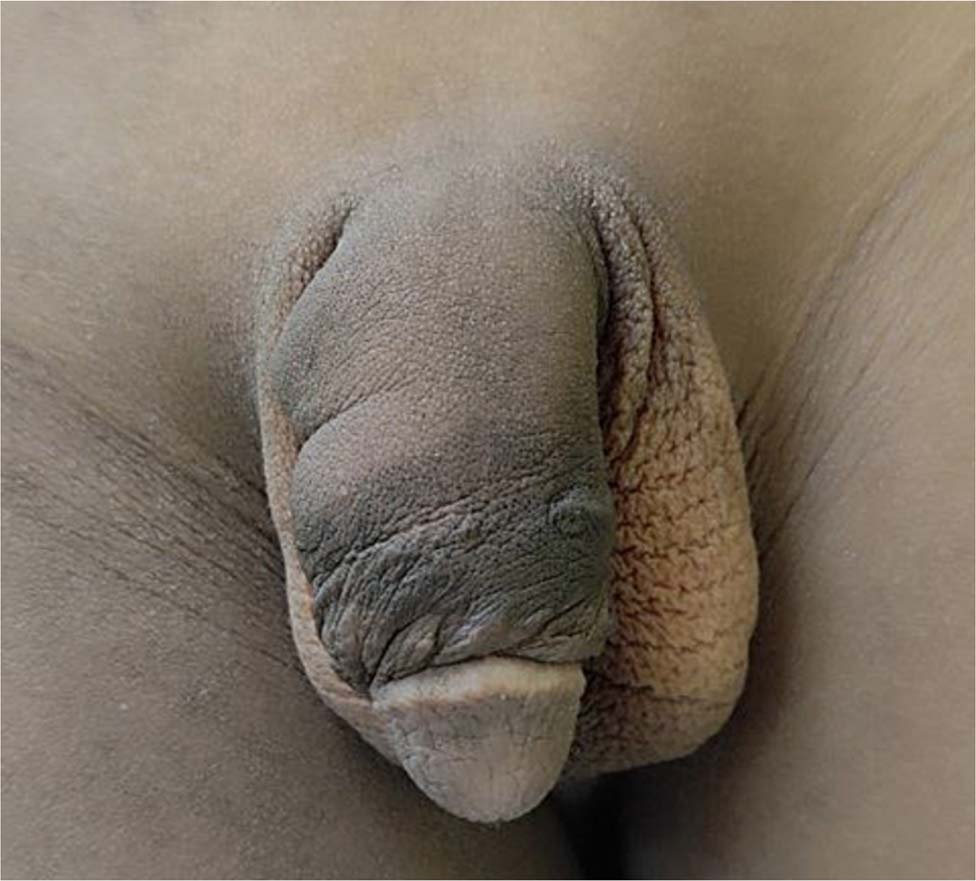
An image at six-month postoperation showing a healed and corrected congenital penile torsion.

Residual torsion was addressed with skin overcorrection by suturing the penile skin against the direction of rotation using PDS 3/0 (Fig. 5). Postoperatively, the patient was discharged after three days with a clean and dry surgical site. Follow-up at two weeks, one month, and six months confirmed a well-corrected penile alignment without complications (Figs 6–8).

## Discussion

Congenital penile torsion, though rare, poses significant psychosocial and functional challenges if left uncorrected. This case highlights a surgical approach that is simple, effective, and adaptable to resource-limited settings. The Dartos flap technique, widely used in urethroplasty for hypospadias repair, demonstrated excellent outcomes in this case without postoperative complications. Compared to other methods such as corporopexy and corporeal plication [2], the Dartos flap approach is less invasive and associated with lower complication rates.

Several techniques have been described to correct penile torsion, but none of them has gained consensus as an optimal, ideal, and versatile technique. Those techniques vary from simple degloving of the penis with repositioning of the penile skin to more complex techniques involving the corporeal tissue depending with the degree of torsion. Correction of penile shaft skin torsion by skin degloving and realignment can be sufficient for correction of mild torsion of <90°. [3–5]. However, when it comes to severe cases with rotation of 90° or more like in our case, other procedure can be done such as suturing the tunica albuginea to the periosteum of the pubis (corporopexy) which is more invasive with more postoperative complications, or diagonal corporeal plication sutures parallel to and away from the neurovascular bundle. As compared to other surgical techniques, the technique used in the index presentation was not associated with any postoperative complications. Other techniques have been significantly associated with higher risks of postoperative complications [3]).

Underreporting of penile torsion cases contributes to the scarcity of robust epidemiological data and surgical guidelines, particularly in LMICs. By documenting this case, we underscore the feasibility and success of using the Dartos flap rotation for isolated penile torsion, even in older children, thus filling a gap in the literature. The technique also avoids complex procedures such as tunica albuginea suturing, which may involve a higher risk of complications [3, 6].

Despite the rarity of congenital penile torsion, its surgical correction offers significant benefits. In this case, the Dartos flap technique proved effective for correcting severe torsion while preserving functionality and esthetics.

Other techniques, such as tunica albuginea suturing, have been reported to be used in other settings with complicated cases. As compared to other surgical techniques, the technique used in the index presentation was not associated with any postoperative complications. Other techniques have been significantly associated with higher risks of postoperative complications [3, 7].

## Conclusion

Congenital penile torsion is a rare and underreported condition that warrants surgical intervention in severe cases. The Dartos flap rotation technique offers a reliable and straightforward corrective method with excellent outcomes and minimal risk of complications. This case report supports the use of this technique as a viable option for managing congenital penile torsion, particularly in resource-constrained settings.

## Data Availability

Not applicable for this case report.
